# Environmental heterogeneity affecting spatial distribution of phytoplankton community structure and functional groups in a large eutrophic lake, Lake Chaohu, China

**DOI:** 10.1007/s11356-023-28043-5

**Published:** 2023-06-06

**Authors:** Huijuan Cao, Kun Zhang, Daogui Deng, Huiying Qi, Jun Li, Yaqin Cao, Qide Jin, Yajie Zhao, Yeping Wang, Zhou Wu, Xinyue Li, Ying Li

**Affiliations:** Anhui Key Laboratory of Resource and Plant Biology, School of Life Sciences, Huaibei Normal University, Huaibei, 235000 China

**Keywords:** Lake Chaohu, Phytoplankton functional groups, Environmental heterogeneity, Non-metric multidimensional scaling analysis

## Abstract

The growth and development of phytoplankton are influenced by physico-chemical parameters, which can also affect the spatial distribution of phytoplankton community structure. However, it is unclear whether environmental heterogeneity caused by multiple physico-chemical factors can affect the spatial distribution of phytoplankton and its functional groups. In this study, we investigated the seasonal variation and spatial distribution of phytoplankton community structure and its relationships with environmental factors in Lake Chaohu from August 2020 to July 2021. We recorded a total of 190 species from 8 phyla, which were divided into 30 functional groups, including 13 dominating functional groups. The average annual phytoplankton density and biomass were (5.46 ± 7.17) × 10^7^ cells/L and 4.80 ± 4.61 mg/L, respectively. The density and biomass of phytoplankton were higher in summer ((14.64 ± 20.34) × 10^7^ cells/L, 10.61 ± 13.16 mg/L) and autumn ((6.79 ± 3.97) × 10^7^ cells/L, 5.57 ± 2.40 mg/L), with the M and H2 of dominant functional groups. The dominant functional groups were N, C, D, J, MP, H2, and M in spring, whereas functional groups C, N, T, and Y dominated in winter. The distribution of phytoplankton community structure and dominant functional groups exhibited significant spatial heterogeneity in the lake, which was consistent with the environmental heterogeneity of the lake and could be classified into four locations. Location I had higher phytoplankton density and biomass than the other three locations. Additionally, dominant functional groups M, C, and H2 were present throughout the lake, and all 13 dominant functional groups were observed in Location II. Our findings suggest that environmental heterogeneity is a key factor influencing the spatial distribution of phytoplankton functional groups in Lake Chaohu.

**Supplementary Information:**

The online version contains supplementary material available at 10.1007/s11356-023-28043-5.

## Introduction

Phytoplankton is the primary producer in aquatic ecosystem, and their growth and reproduction are limited by environmental factors including water temperature, nutrient levels, and light availability (Vanni 1987; Deng et al. 2007; Rakočević 2018; Ma et al. 2022). The fluctuations of physico-chemical factors in the water body can be reflected by phytoplankton, and changes in its community structure can indicate the intensity of the top-down effect. As a result, phytoplankton is often utilized as a crucial indicator for monitoring freshwater water quality (Haque et al. 2021; Luo et al. 2022; Chen et al. 2023; Su et al. 2023).

The composition of phytoplankton communities is influenced by the abiotic factors (e.g., lake habitats, water temperature, and nutrients) and biotic factors (e.g., zooplankton grazing and fish predation) together, which is able to respond quickly to water environmental changes (Chen et al. 2013; Napiórkowska-Krzebietke 2017; Zhang et al. 2019). The heterogeneity of these environmental factors can result in a diverse phytoplankton community structure. However, there is a certain degree of discrepancy between the habitat characteristics represented by phytoplankton classification in terms of community structure, cell abundance, and biomass, which poses a challenge for accurately assessing aquatic eutrophication and ecological health (Reynolds et al. 2002; Wang et al. 2023a). Moreover, the utilization of phytoplankton cell density for establishing diversity indices can underestimate species richness, which may lead to poor evaluation of the ecological status of aquatic bodies (Su et al. 2023).

Several researchers have proposed that phytoplankton with similar morphology, physiology, and survival strategies can be grouped together into functional groups in similar aquatic habitats. In contrast, different types of phytoplankton form distinct functional groups. (Reynolds et al. 2002; Padisák et al. 2009). The introduction of phytoplankton functional groups has overcome the limitations of traditional classification methods, which fail to accurately reflect the ecological characteristics of habitats. This approach has several advantages in ecological research and application (Devercelli 2006; Varol 2019). Yan et al. (2023) found that the phytoplankton functional group responded more strongly to environmental factors than the phytoplankton species in Lake Dongting.

Lake Chaohu, located in the western zone near Hefei city, receives nutrient inflow from several upper inlet rivers, namely, the Hangbuhe River, Nanfeihe River, Baishitianhe River, Paihe River, Shiwulihe River, and Zhaohe River. This nutrient inflow has led to the eutrophication of Lake Chaohu over the past 40 years. Additionally, the presence of a phosphate mine on the northeast shore of the lake has contributed to the maintenance of high phosphorus levels in the lake over a long period (Deng et al. 2007; Liu and Meng 1988; Jiang et al. 2014). The cyanobacterial bloom (especially *Microcystis* and *Dolichospermum*) in Lake Chaohu experienced a complex and long-term process. It initially appeared in the western zone of the lake and subsequently spread to other regions before occurring throughout the entire lake. However, the western zone of the lake has experienced stronger cyanobacterial blooms than the eastern zone for more than 30 years (Liu and Meng 1988; Deng et al. 2007; Jiang et al. 2014; Li et al. 2017; Zhang et al. 2018). This pattern may be attributed to the environmental heterogeneity in Lake Chaohu.

Furthermore, there have been significant alterations in the fish yield and composition of Lake Chaohu. In 1952, the annual fish yield was recorded at 3500 t, with 41.1% of phytophagous fish species such as *Hypophthalmichthys molitrix* and *Aristichthys nobilis* and 56.2% of zooplanktivorous fish species such as *Coilia ectenes*. Since the 1970s, the proportion of *C. ectenes* remained at high level (80% in 1973, 61.4% in 1984, and 75% in 2002) (Wang 1987; Lake Chaohu Fishery Administration Committee, unpublished data). The annual fish yield increased approximately 8000 t in 2002 (Deng et al. 2007). At present, the small zooplanktivorous *C. ectenes* and *Neosalanx taihuensis* are predominant (Liang et al. 2022), whereas large-bodied crustacean zooplanktons (e.g., *Daphnia*) are rare in warm seasons (Deng et al. 2008; Li et al. 2023). Therefore, the dominance of zooplanktivorous fish and small crustacean zooplankton can favor the development of filamentous or colony cyanobacteria (especially *Microcystis* and *Dolichospermum*) in Lake Chaohu, affecting the succession of phytoplankton functional groups. However, fishing ban at the whole lake has been executed since January 2020 in Lake Chaohu. This way of comprehensive fishing ban in a large lake (with an area of 780 km^2^) is rare in the world, and its impact mechanism on plankton remains unclear.

The aim of this study is to assess the impact of environmental heterogeneity on the temporal fluctuations and spatial patterns of phytoplankton functional groups in a persistent high-phosphorus lake and to explore the response mechanism of phytoplankton community structure and dominant functional groups under high-intensity fish predation in Lake Chaohu.

## Materials and methods

### Study stations

**Fig. 1:**
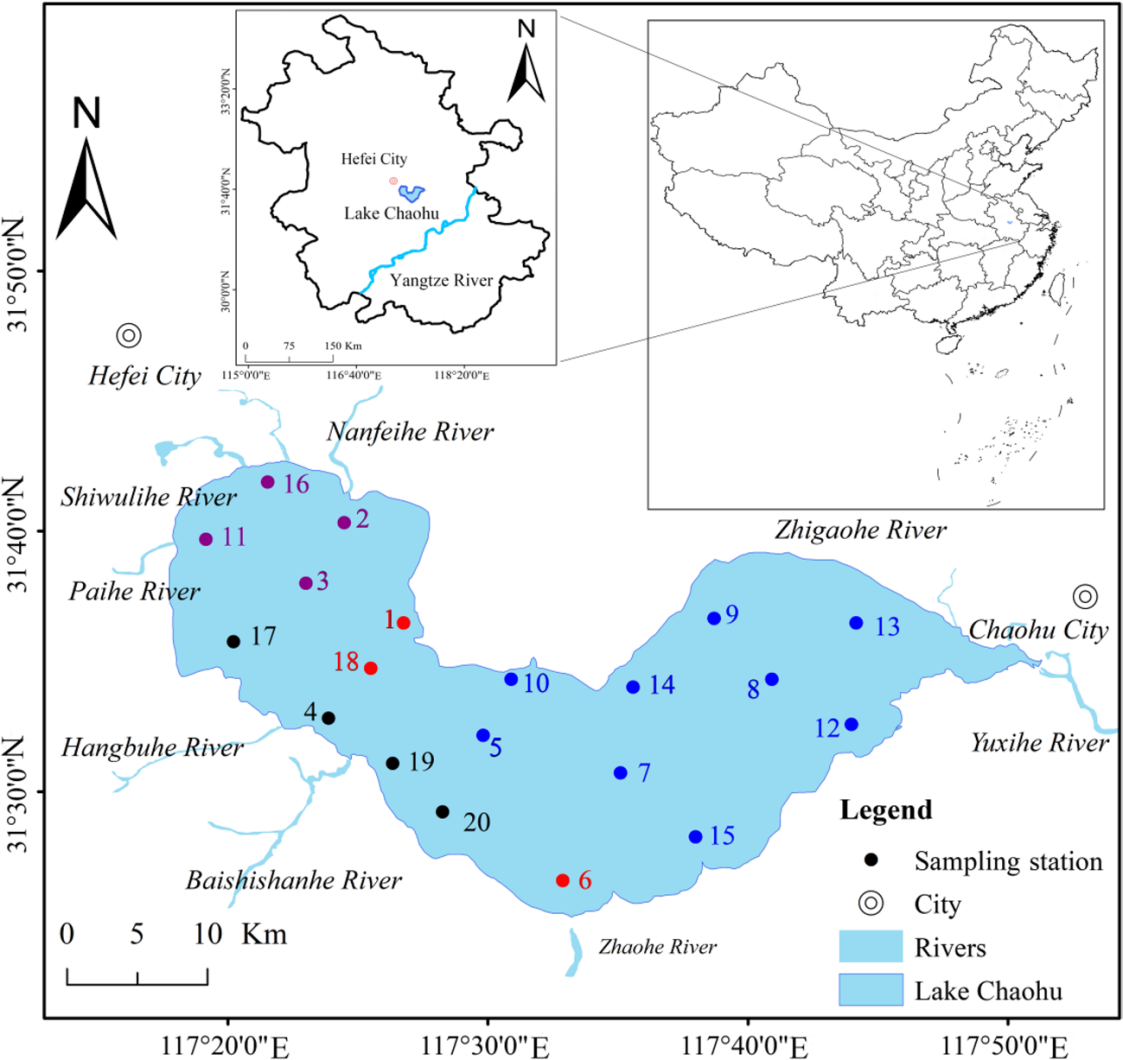
Distribution of sampling stations in Lake Chaohu

Lake Chaohu (117° 18′–117° 50′ E, 31° 25′–31° 43′ N) is located in the middle and lower reaches of the Yangtze River, China, with an area of 780 km^2^. It is a subtropical shallow lake and has the functions of climate regulation, recreation, and fishery resources (Deng et al. 2007; Jiang et al. 2014). Lake Chaohu has gradually become a semi-enclosed lake since a sluice was built in 1960. Phytoplankton community was monthly investigated at 20 sampling stations in Lake Chaohu between August 2020 and July 2021 (Fig. 1). Stations (1–4, 11, and 16–18) were situated in the more eutrophic western region of the lake, whereas stations (5–10, 12–15, and 19–20) were in the less eutrophic eastern zone of the lake.

### Physico-chemical parameters in Lake Chaohu

pH was measured by a portable pH meter (HACHQ11D, USA); dissolved oxygen (DO) and water temperature (WT) were determined by a portable dissolved oxygen meter (HANNA, Italy) in the field. Water depth (WD) and transparency (SD) were determined by the Secchi disk at each sampling station.

Water samples for nutrient measurements were a mixture of sub-samples from the surface to the bottom at 1-m or 2-m intervals. One-liter mixed water sample was taken to determine the nitrogen and phosphorus concentrations in the laboratory. Total nitrogen (TN) was analyzed with the alkaline persulfate oxidation method (Huang 1999). Total phosphorus (TP) and dissolved total phosphorus (DTP) were analyzed with the persulfate oxidation method and molybdenum antimony anti-color development spectrophotometric method (SEPB 2002). Before determining dissolved total phosphorus, water samples were filtered by Whatman GF/C filter (Lu et al. 2010; Yang et al. 2019).

### Collection, identification, and counting of crustacean zooplankton

Crustacean zooplankton samples were collected with a 5-L modified Patalas bottle sampler. A total of 30-L mixed water samples at each sampling site were obtained from the surface to the bottom at 0.5-m or 1-m intervals. Cladocerans and copepods were filtered with a 25# plankton net (64 μm) and then fixed with 4% formaldehyde solution. The crustacean zooplankton were identified according to the methods of Jiang and Du (1979) and Sheng (1979).

### Phytoplankton analysis

Each phytoplankton sample was a mixture of sub-samples taken from the surface to bottom with a 5-L modified Patalas bottle sampler at 1-m or 0.5-m intervals. Phytoplankton sample (1 L) was collected from a mixed sub-sample at each station and then was fixed with 10 mL of Lugol’s iodine solution and sedimented for 48 h prior to counting under a microscope. The remains were stored in a 50-mL plastic bottle and preserved in the dark in the laboratory. The concentrated solution (0.1 mL) was absorbed after thoroughly shaking and placed in a counting box under a microscope (Olympus CX31) and then observed and counted. For each sample, it was counted twice, and about 100 fields were observed each time. The average value twice was regarded as the final result. It was valid of the error twice which was within 15% (Wu et al. 2022a, b; Wang et al. 2023a, b).

The cell density of each phytoplankton species was calculated in a formula: *N* = *n* × *A* × *V*_s_/(*A*_c_ × *V*_a_), where *N* is the cell density of a species in a sample (× 10^5^ cells/L); *A* is the total number of fields in the microscope; *A*_c_ is the number of fields observed; *V*_a_ is the volume of the counting box (mL); *V*_s_ is the volume (mL) concentrated by 1-L original water sample precipitation; and *n* is the cell number of the counted phytoplankton.

Phytoplankton species were identified according to the methods of Hu (1980), Hu and Wei (2006), and Zhang et al. (2018). Phytoplankton density and biomass (wet weight) were calculated according to the method of Zhang and Huang (1991).

### Statistical analysis

The biomass of phytoplankton functional groups can explain the dynamics of phytoplankton community structure and response to environmental characteristics (Hu et al. 2015). The phytoplankton functional groups are classified according to the methods of Reynolds et al. (2002) and Padisák et al. (2009), and those with a relative biomass above 5% are defined as dominant functional groups (Sevindik et al. 2014). The formula is *P*_*i*_ = *n*_*i*_/*N*, in which *n*_*i*_ is the biomass of the *i*th functional group, and *N* is total biomass of phytoplankton.

The dominant species of phytoplankton are determined based on the dominance value (*Y*) of each species. The formula is *Y* = (*n*_*i*_/*N*)*f_*i*_, in which *n*_*i*_ stand for the individual number of *i* species, *N* is total individual number of phytoplankton, and *f*_*i*_ is the frequency of occurrence of *i* species in the sample. When *Y* ≧ 0.02, phytoplankton is regarded as dominant species (Jiang et al. 2014).

Detrended correspondence analysis (DCA) is performed to analyze the relationships between dominant functional groups and environmental factors, and the maximum gradient length is found to be less than 3. Therefore, redundancy analysis (RDA) and variation partitioning analysis (VPA) are determined to analyze the relationships between dominant functional groups of phytoplankton and environmental factors using Canoco 5.0 software (Liu et al. 2019). Non-metric multidimensional scale analysis (NMDS) is employed to analyze spatial distribution of dominant functional groups of phytoplankton and environmental factors (including TN, TP, TDP, pH, DO, WD, and SD) in Lake Chaohu using Past 4.0 software (Zhang and Kong 2015; Qu et al. 2022).

During statistic analysis, the data are transformed by Log (*x* + 1) before analysis except pH. Two-way ANOVA was employed to examine the influence of locations (I, II, III, and IV), seasons (spring, summer, autumn, and winter), and their combination on the physico-chemical factors, phytoplankton density, and biomass in Lake Chaohu using SPSS 26.0 software.

## Results

### Temporal-spatial variations of physico-chemical factors in Lake Chaohu

The maximum water temperature appeared in August (30.95 ± 0.62 °C) whereas the minimum value was in January (2.67 ± 0.34 °C) (Table 1; Supplementary Table 1). The maximum DO (10.51 ± 0.89 mg/L) appeared in December, and the lowest annual average value of DO was in station 2 (6.74 ± 1.70 mg/L). Both the highest TN concentration (2.14 ± 2.69 mg/L) and TP concentration (0.42 ± 0.07 mg/L) occurred in August, and the annual maximum average value of TN concentration (2.76 ± 2.76 mg/L) and TP concentration (0.40 ± 0.45 mg/L) all appeared in station 11; the annual mean minimum values of TN (0.73 ± 0.38 mg/L) and TP (0.20 ± 0.05 mg/L) appeared all at stations 15 and 8, respectively. The maximum DTP concentration appeared in October (0.11 ± 0.02 mg/L) whereas the minimum value was in January (0.02 ± 0.01 mg/L). The average annual N/P ratio was 6.08 ± 3.25, with a peak value of 16.20 ± 10.85 in January. Water depth was relatively stable in almost months, but it reached the peak (5.66 ± 0.56 m) in August after heavy rainfall whereas the minimum value (2.89 ± 0.59 m) was in March. The maximum SD appeared in August (0.51 ± 0.09 m) whereas the lowest value was in December (0.19 ± 0.03 m). pH (8.37 ± 0.28) was alkaline during the sampling period; the maximum pH (9.42) appeared at station 16 in September.

**Table 1:** Ranges and average values of physicochemical parameters in Lake Chaohu

Parameters	Range	Average values
Water temperature (℃)	2.67–30.95	19.00 ± 8.65
Dissolved oxygen (mg/L)	6.22–10.51	7.45 ± 1.13
Total nitrogen (mg/L)	0.40–2.14	1.37 ± 0.59
Total phosphorus (mg/L)	0.14–0.42	0.25 ± 0.08
Dissolved total phosphorus (mg/L)	0.02–0.11	0.05 ± 0.02
N/P	2.02–16.20	6.08 ± 3.25
Water depth (m)	2.74–5.66	3.59 ± 0.81
Transparency (m)	0.19–0.51	0.29 ± 0.01
pH	7.90–8.85	8.37 ± 0.28

**Fig. 2:**
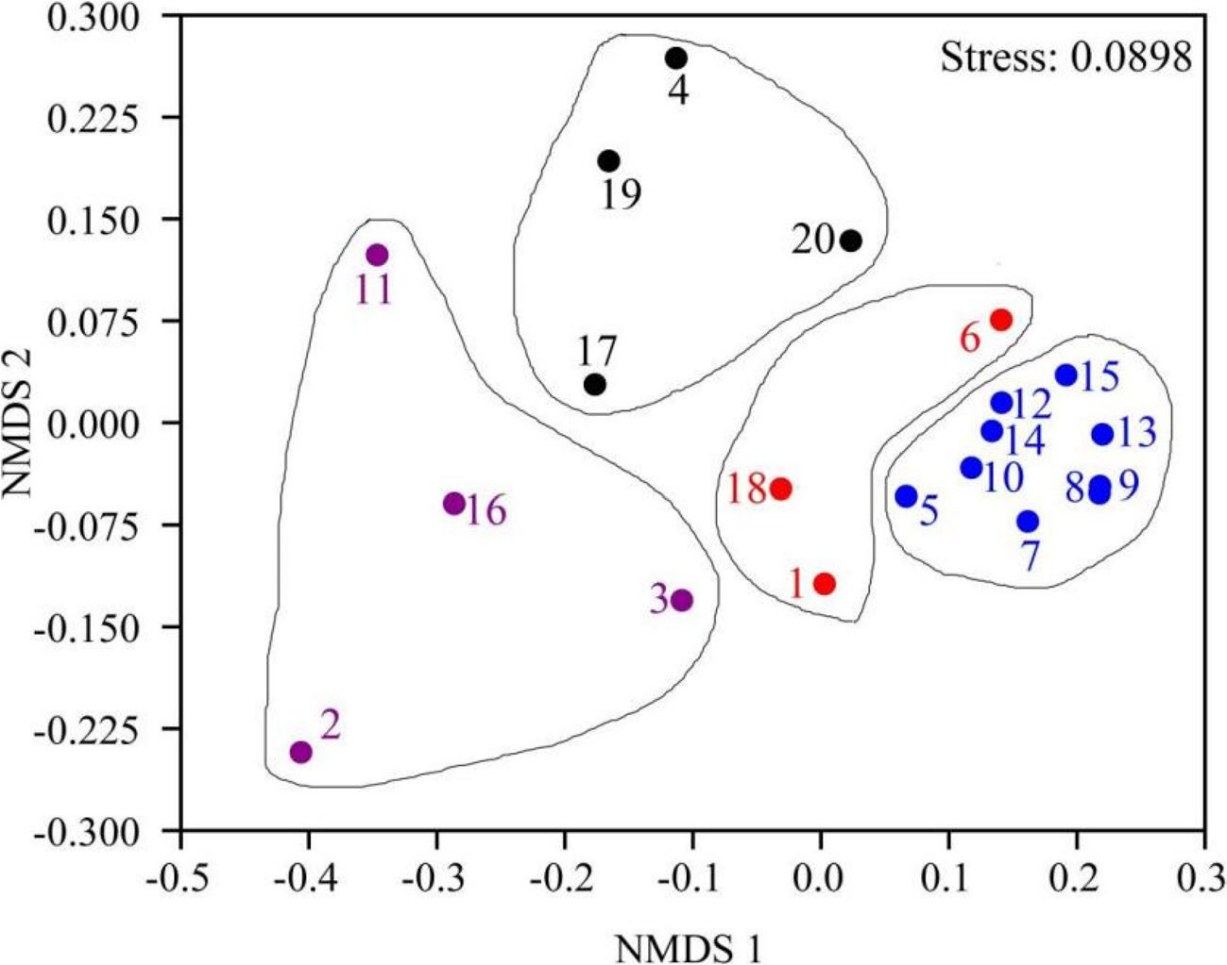
Spatial heterogeneity of environmental factors in Lake Chaohu based on non-metric multidimensional scale analysis (NMDS)

Non-metric multidimensional scaling analysis (NMDS) shows there is a clear spatial heterogeneity in the physico-chemical properties of the lake, and it can be divided into four locations (Fig. 2). Both Location I (stations 2, 3, 11, and 16) and Location II (stations 4, 17, 19, and 20) locate in the western zone of the lake where the inlets of several polluted rivers are located. Higher nitrogen and phosphorus concentrations during the sampling period appeared in Location I. Location IV (stations 5, 7, 8, 9, 10, 12, 13, 14, and 15) is located in the eastern of the lake, with lower nutrient levels. Location III (stations 1, 6, and 18) is located between two lake areas.

### Temporal-spatial variations of crustacean zooplankton in Lake Chaohu

In Lake Chaohu, the density of crustacean zooplankton shows obvious tempo-spatial variations. The annual average density of crustacean zooplankton is 58.91 ± 63.72 ind./L, with the range of monthly average density from 6.94 ± 3.59 ind./L to 236.15 ± 98.82 ind./L. The maximum value (236.15 ± 98.82 ind./L) appears in August, in which the dominant species are *Bosmina longirostris*, *Ceriodaphnia cornuta*, *Chydorus sphaericus*, *Diaphanosoma brachyurum*, *Moina micrura*, *Thermocyclops* sp., and *Mesocyclops leuckarti*, and the minimum value (6.94 ± 3.59 ind./L) occurs in April, in which the dominant species are *Sinocalanus dorrii*, *Limnoithona sinensis*, and *B. longirostris*. Spatially, the maximum (134.98 ± 32.47 ind./L) of crustacean zooplankton occurs in Location I in summer, in which *B. longirostris*, *C. cornuta*, *C. sphaericus*, *L. sinensis*, and *Thermocyclops* sp. dominate, and the minimum density (16.54 ± 7.17 ind./L) appears in Location IV in spring, in which *B. longirostris*, *S. dorrii*, *Bosmina fatalis*, and *L. sinensis* dominated.

### Temporal-spatial variations of phytoplankton community structure in Lake Chaohu

#### Temporal-spatial variations in phytoplankton density and biomass

**Fig. 3:**
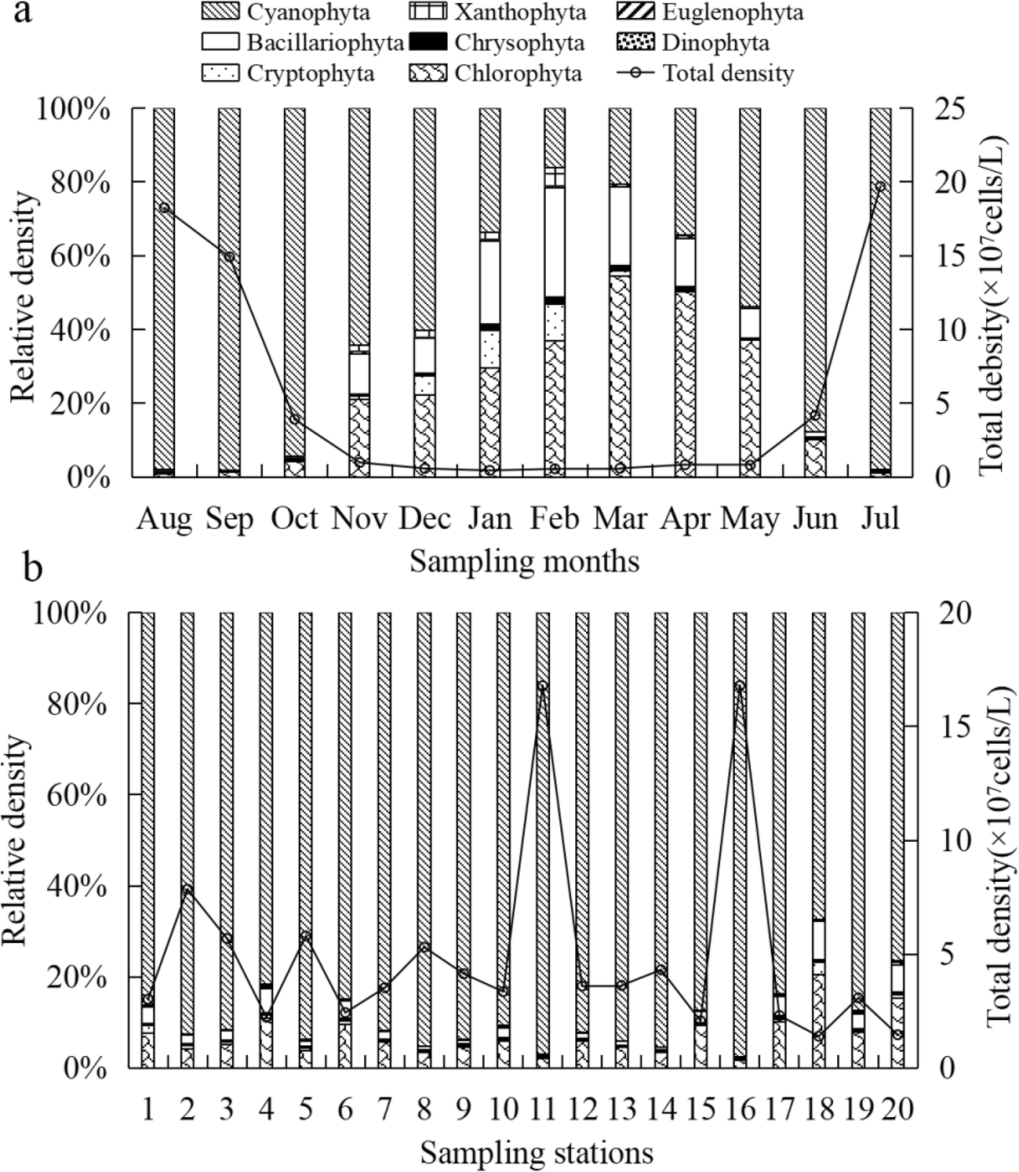
Tempo-spatial variations on relative densityand total density of phytoplankton in Lake Chaohu during 2020–2021, **a**: show monthly average relative density of each phylum and total cell density of phytoplankton at twenty sampling stations in Lake Chaohu, **b**: show annual average relative density of each phylum and total cell density of phytoplankton at each sampling station in Lake Chaohu

The density of phytoplankton vary from 0.43 × 10^7^ cells/L to 20.91 × 10^7^ cells/L, with an annual mean value of (5.46 ± 7.42) × 10^7^ cells/L in Lake Chaohu. Phytoplankton density shows a significant seasonal difference (*P* < 0.001), in which it increases in spring, reaches the peak in summer, and declines in autumn and winter (Fig. 3a). Chlorophyta become the dominant group in spring, which account for 47% of total phytoplankton density in spring, followed by Cyanophyta (38%) and Bacillariophyta (14%). Cyanophyta is the dominant group in summer (97%) and autumn (96%). In winter, Cyanophyta (37%), Chlorophyta (29%), and Bacillariophyta (20%) are predominant. The spatial distribution of phytoplankton density have also an obvious difference (Fig. 3b). The first two peaks of phytoplankton density are found at station 16 ((16.80 ± 0.24) × 10^7^ cells/L) and station 11 ((16.79 ± 0.24) × 10^7^ cells/L), and the lowest density appears at station 20 ((1.46 ± 0.16) × 10^7^ cells/L). The relative density of Cyanophyta in each sampling station is more than 65% among total phytoplankton density, followed by Chlorophyta and Bacillariophyta. The highest relative density of Cyanophyta occurs at station 16 (97.47%). The peak of relative density of Chlorophyta and Bacillariophyta both appear at station 18 (20.60%) and station 20 (15.31%), respectively. Two-way ANOVA shows that the effects of seasonal variation (*F* = 65.009, *P* < 0.001) and spatial distribution (*F* = 6.833, *P* < 0.001) on phytoplankton density are all very significant (Supplementary Table 1).

**Fig. 4:**
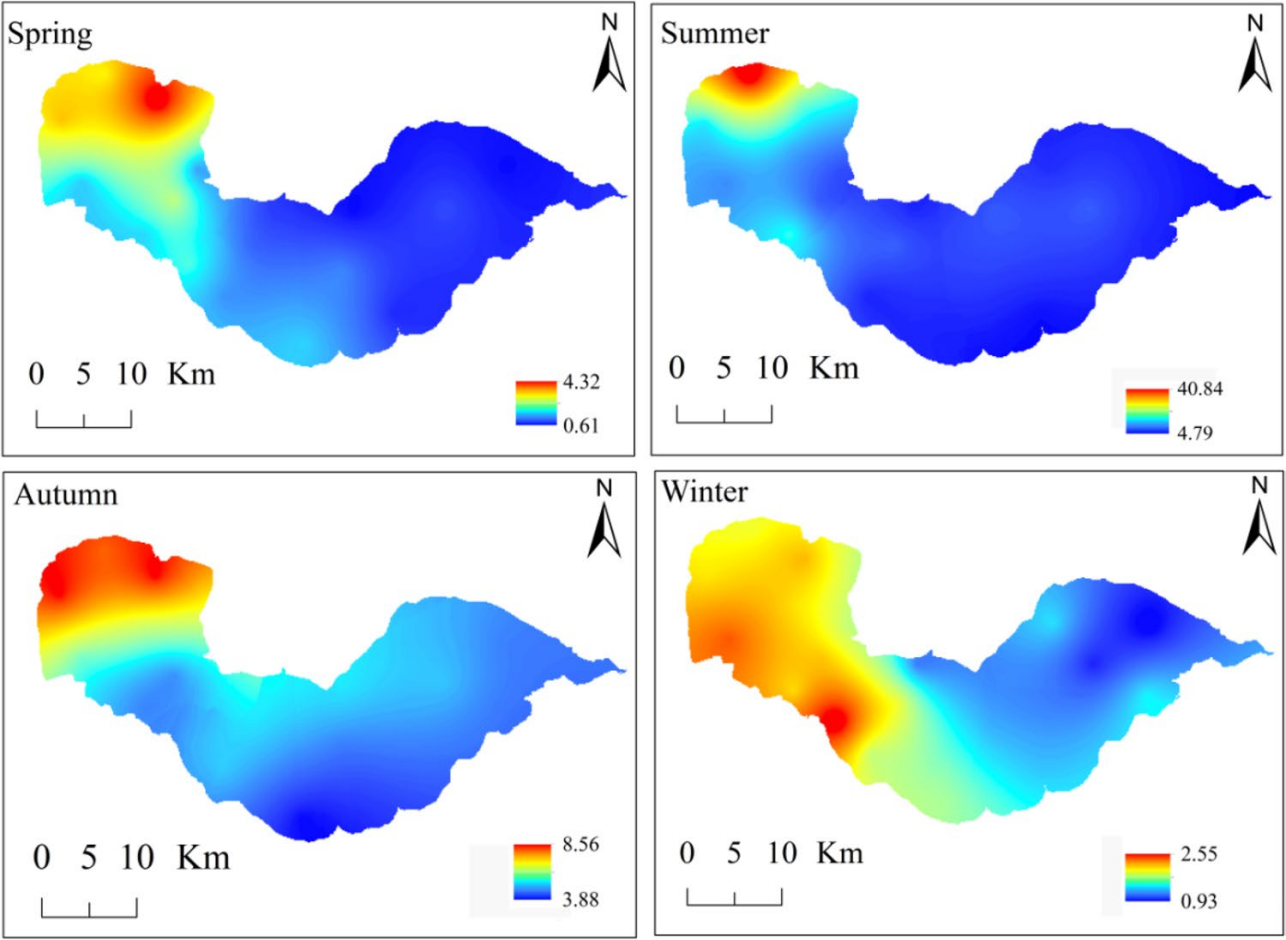
Tempo-spatial variations of seasonal average phytoplankton biomass (mg/L) in Lake Chaohu during 2020–2021. Values represent the minimum and maximum values of average phytoplankton biomass in four seasons. The interpolation map was constructed by ArcGIS 10.4 software using Kriging interpolation method

The monthly average phytoplankton biomass ranges from 1.27 to 15.20 mg/L, with an annual mean value of 4.80 ± 4.61 mg/L in Lake Chaohu. The maximum biomass (15.20 ± 25.67 mg/L) occurs in July whereas the minimum value (1.27 ± 0.47 mg/L) appears in December. Phytoplankton biomass shows obvious temporal and spatial variations (Fig. 4). The lowest seasonal average biomass of phytoplankton is in spring (1.60 ± 1.00 mg/L), which is dominated by Bacillariophyta (0.68 ± 0.15 mg/L) and Chlorophyta (0.61 ± 0.07 mg/L), and *Cyclotella meneghiniana* (*Y* = 0.175) is the dominant species. It reached the peak (10.61 ± 13.16 mg/L) in summer, in which the proportion of Cyanophyta is 81%, and *Microcystis* sp. (*Y* = 0.566) and *Dolichospermum* sp. (*Y* = 0.147) are the dominant species. In autumn, it is 5.57 ± 2.40 mg/L, with a relative biomass of 69% Cyanophyta, and then, it is 1.69 ± 0.48 mg/L in winter, with the proportion of 44% Bacillariophyta and 22% Chlorophyta. Spatially, phytoplankton biomass in the western zone of the lake is higher than that in the eastern zone in four seasons (Fig. 4). The average annual phytoplankton biomass at stations 11 (11.11 ± 19.53 mg/L) and 16 (14.57 ± 36.40 mg/L) is obviously higher than that at the other stations. Two-way ANOVA shows that the effects of seasonal variation (*F* = 44.716, *P* < 0.001), spatial distribution (*F* = 11.927, *P* < 0.001), and their combination (*F* = 2.265, *P* < 0.01) on phytoplankton biomass are all very significant (Supplementary Table 1).

### Temporal-spatial variations of phytoplankton functional groups in Lake Chaohu

**Fig. 5:**
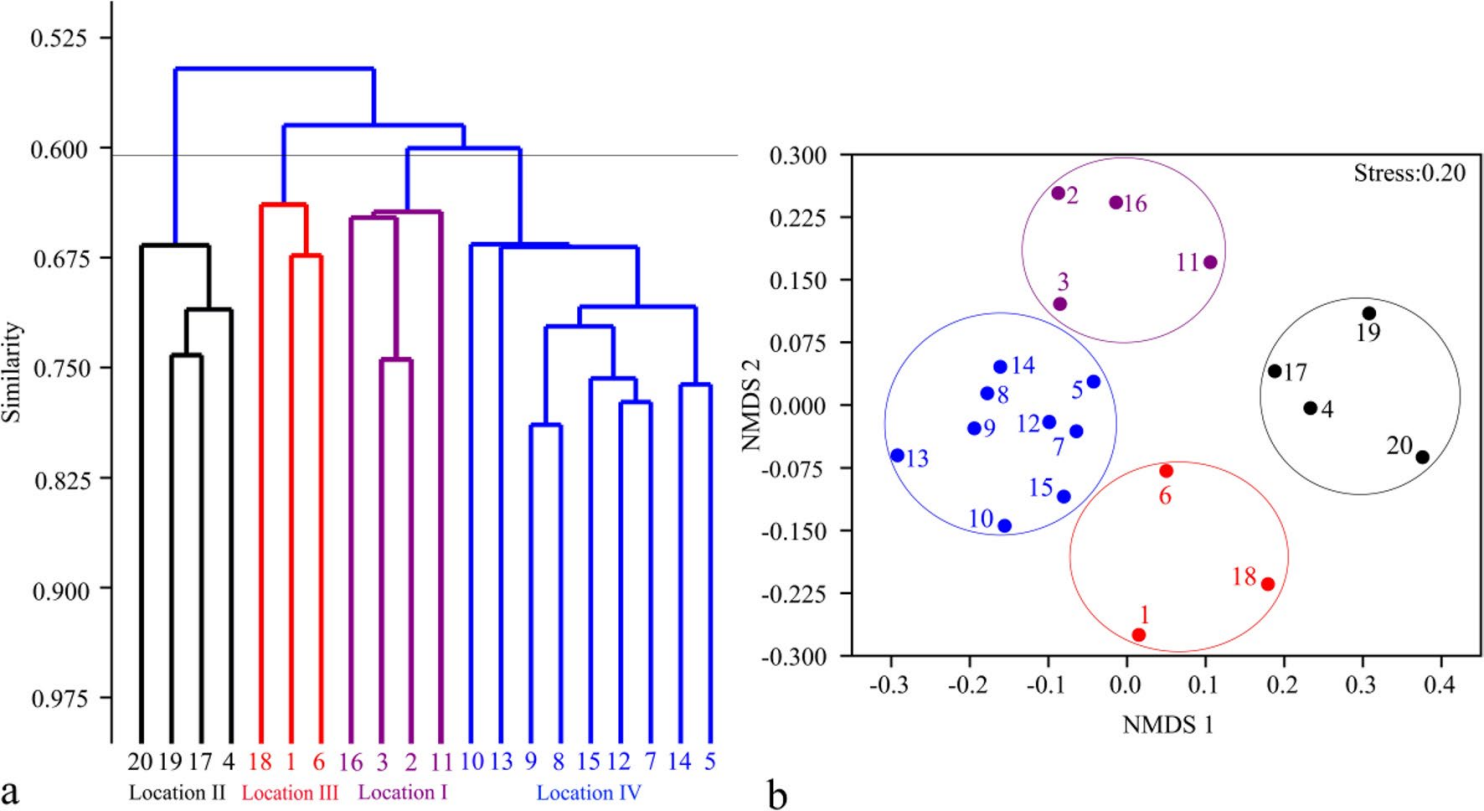
Spatial distribution of the biomass of dominant functional groups of phytoplankton in Lake Chaohu based on clustering (**a**) and Non-metric multidimensional scale (**b**) analysis

**Fig. 6:**
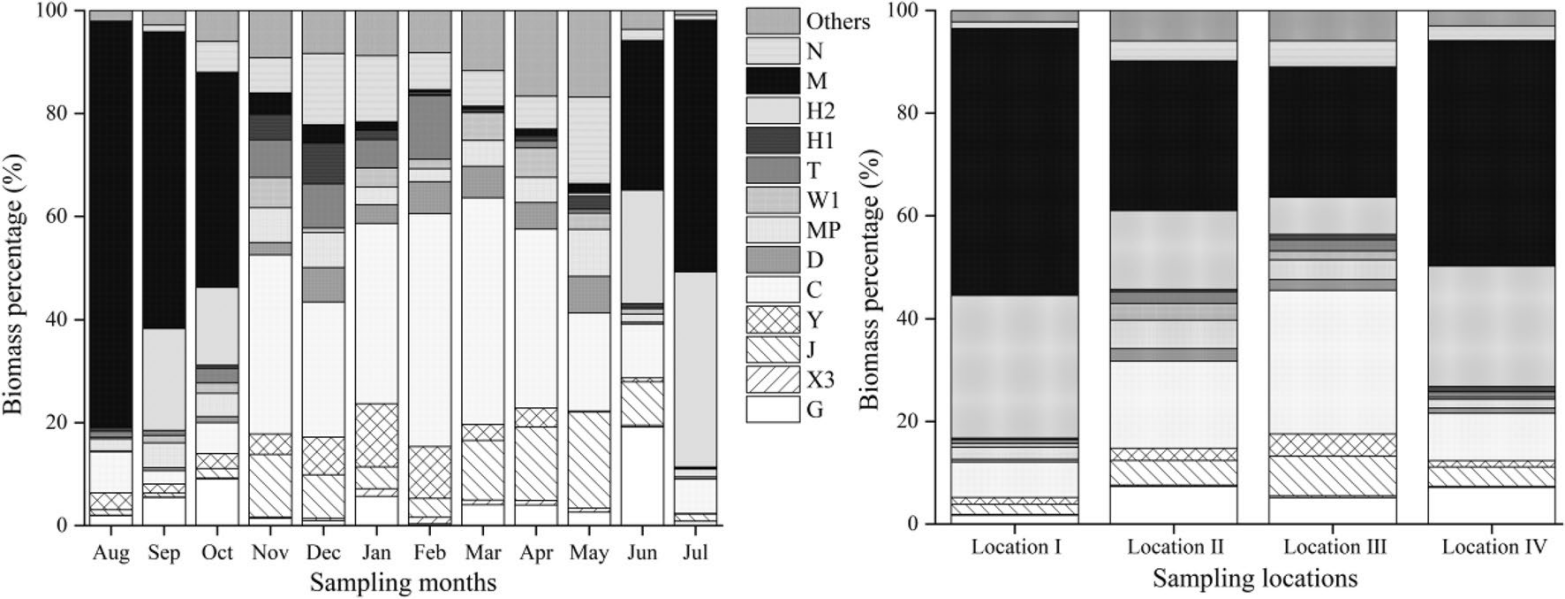
Temporal and spatial dynamics of the biomass percentage of dominant functional groups of phytoplankton in Lake Chaohu

A total of 190 species of phytoplankton belonging to 8 phyla are identified during the study period (Supplementary Table 2). In terms of species number, Chlorophyta (94 species) ranks the first, followed by Bacillariophyta (39 species), Cyanophyta (26 species), Euglenophyta (14 species), Chrysophyta (6 species), Cryptophyta (4 species), Dinophyta (4 species), and Xanthophyta (3 species). Thirty functional groups of phytoplankton are detected in Lake Chaohu, among which thirteen functional groups are dominant, according to the traits of these species (Table 2). Spatially, based on the biomass of dominant functional groups, phytoplankton are clustered into four locations (I, II, III, and IV) in Lake Chaohu, with a 60–67% Bray–Curtis similarity, namely, Location I (stations 2, 3, 11, 16), Location II (stations 4, 17, 19, 20), Location III (stations 1, 18, 6), and Location IV (stations 5, 7, 8, 9, 10, 12, 13, 14, 15) (Fig. 5a). The two-dimensional arrangement of NMDS shows also that phytoplankton are divided into four locations (Fig. 5b). Thirteen dominant functional groups all present in four locations of the lake, but each dominant functional group shows the difference in different locations. Temporally, functional groups C, D, J, N, MP, H2, and M are dominant in spring, accounting for 71.26% of the total biomass, and the highest relative biomass of both D (7.04%) and MP (9.10%) appears in May. Functional groups M (55.62%) and H2 (22.05%) are the dominant functional groups in summer, and their highest relative biomass occurred in August and July, respectively. Functional groups C, G, and MP co-existed with H2 and M, accounting for 76.63% of the total biomass together in autumn. In winter, the dominant functional groups are functional groups C, N, Y, and T, and their highest relative biomass occurs respectively in February (45.15%), December (13.86%), and February (12.38%) (Fig. 6).

**Table 2:** The classification of phytoplankton functional groups in Lake Chaohu

FGs	Representative algae species	Habitat environment	*F* factor
A	*Urosolenia* spp.	Clear, deep and base poor lakes	5
C	*Melosira* spp., *Cyclotella*, *Asterionella formosa*, *Stephanodiscus* spp.	Mixed, eutrophic small- medium lakes	4
D	*Synedra* spp., *Nitzschia* spp., *Coscinodiscus* spp.	Shallow turbid waters including rivers	2
E	*Dinobryon* spp.	Shallow, base poor lakes or heterotrophic ponds	2
F	*Kirchneriella* spp., *Oocystis* spp., *Oocystis borger*, *Selenastrum* spp., *Selenastrum minutum*, *Quadrigula chodatii*	Clean, deeply mixed meso-eutrophnic lakes	3
G	*Eudorina elegans*, *Pandorina morum*, *Dictyosphaerium pulchellum*,	Nutrient-rich conditions in stagnating water columns	2
H1	*Aphanizomenon flos-aquae*	Eutrophic, both stratified and shallow lakes with low nitrogen content	1
H2	*Dolichospermum* spp.	Oligo-mesotrophic, deep, stratifying lakes or mesotrophic shallow lakes	3
J	*Tetraedron* spp., *Pediastrum biradiatum*, *Scenedesmus* spp., *Crucigenia* spp., *Coelastrum sphaericum*, *Desmodesmus quadricauda*, *Tetrastrum* spp.	Shallow, mixed, highly enriched systems	1
K	*Aphanocapsa* spp., *Aphanothece* spp.	Shallow and nutrient-rich water columns	2
L_M_	*Ceratium hirundinella*	Eutrophic to hypertrophic, small to medium-sized lakes	0
L_O_	*Peridinium* spp., *Merismopedia* spp.	Deep and shallow, oligo to eutrophic, medium to large lakes	1
M	*Microcystis* spp.	Eutrophic to hypertrophic, small to medium sized waterbodies. Sensitive to low light	0
MP	*Navicula* spp., *Surirella* spp., *Oscillatoria* sp., *Pesudanabaena* spp.	Frequently stirred up, in organically turbid shallow lakes	3
N	*Staurastrum* spp., *Cosmarium* spp.	Mesotrophic epilimnia	2
N_A_	*Staurodesmus* spp., *Gonatozygon* spp.	Atelomictic environments at lower latitudes with species sensitive to destratification	4
P	*Closterium* spp., *Fragilaria* spp., *Cymbella* spp.	Eutrophic epilimnia	5
S_1_	*Dactyloccocopsis* spp.	Turbid mixed environments	0
S_2_	*Spiralina* spp., *Phormidium* spp.	Warm, shallow and often highly alkaline waters	2
S_N_	*Raphidiopsis* spp.	Warm mixed environments	0
T	*Tribonema* spp.	Deep, low light	4
T_C_	*Lyngbya* spp.	Eutrophic standing waters, or slow-flowing rivers with emergent macrophytes	1
W_O_	*Chlamydomonas* spp.	Rivers and ponds with extremely highorganic contents, even septic for most aquatic biota	1
W_1_	*Euglena* sp., *Phacus* sp.	Shallow organic lakes	1
W_2_	*Trachelomonnas* sp., *Strombomonas* sp.	Meso-eutrophic shallow lakes	1
X1	*Ankistrodesmus* sp.	Shallow eu-hypertrophic Environments	3
X2	*Chroomonas acuta*, *Chroomonas caudata*	Shallow, meso-eutrophic environments	3
X3	*Schroederia* sp., *Schroederia nitzschioides*, *Ochromonas* sp., *Chlorella vulgaris*	Shallow, well mixed oligotrophic environments	4
Y	*Cryptomona ovata*, *Cryptomona erosa*, *Gymnodinium aeruginosum*	Hydrostatic environment	2
Z	*Actinastrum fluviatile*	Metalimnia or upper hypolimnia of oligotrophic lakes	5

The relative biomass of dominant functional groups in Locations I and IV is predominated by functional groups M (52.01%), H2 (27.70%), and C (6.89%). Functional groups M (29.19%) and C (16.97%) dominate in Location II. Functional groups J, C, H2, and M are the dominant functional groups in Location III, accounting for 68.26% of the total relative biomass. The dominant function groups in Location IV are M (43.95%), H2 (23.38%), and C (9.20%), respectively. In summary, dominant function groups M, H2, and C remain at a high level in space, and their maximum relative biomass is in Location I (52.01%), Location III (27.87%), and Location IV (23.38%), respectively (Fig. 6).

### Relationships between dominant functional groups of phytoplankton and environmental factors

**Fig. 7:**
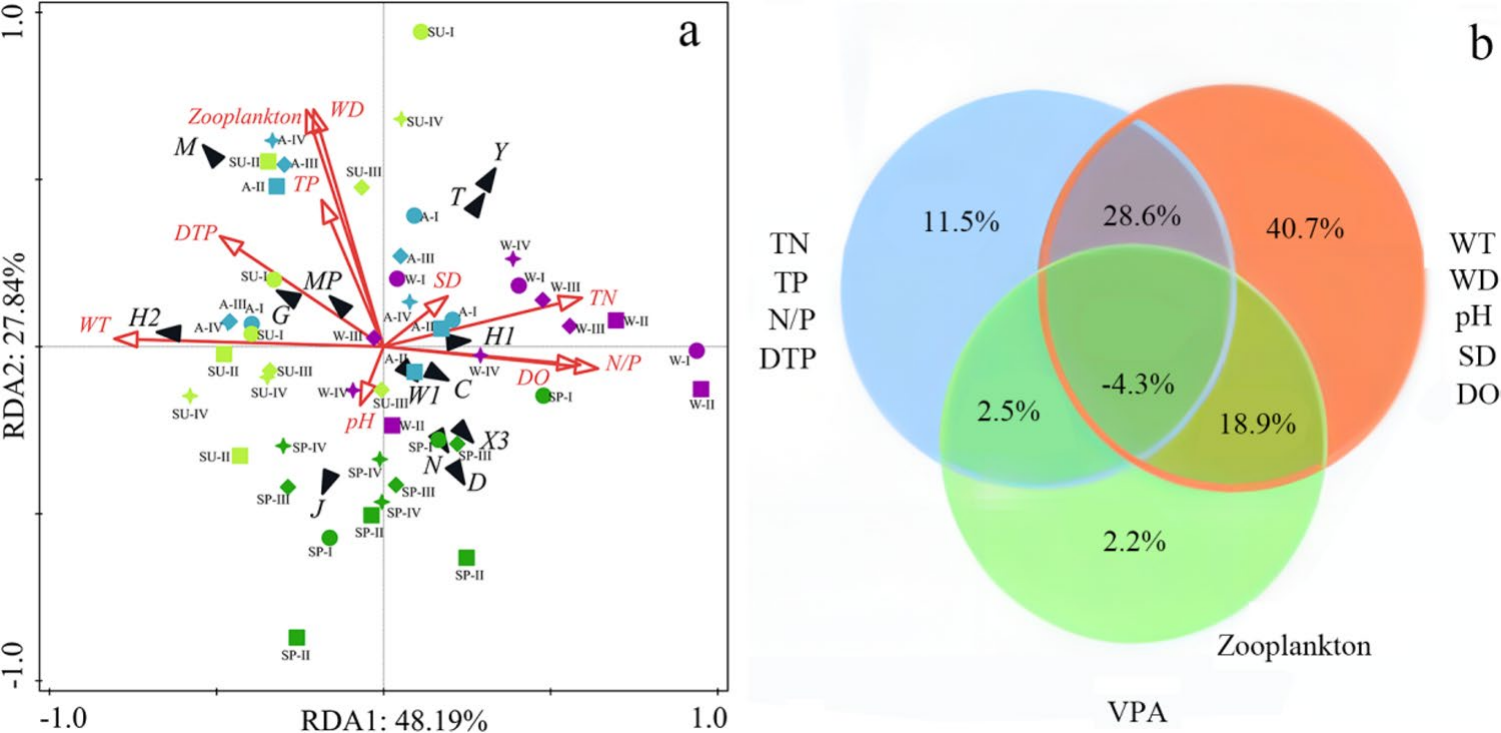
Redundancy analysis (RDA, **a**) and variation partitioning analysis (VPA, **b**) between dominant functional groups of phytoplankton and environmental factors during 2020–2021 in Lake Chaohu, (WT: Water temperature, DO: Dissolved oxygen, TN: Total nitrogen, TP: Total phosphorus, DTP: Dissolved total phosphorus, N/P: TP/TP, WD: Water depth, SD: Transparency. ●: Location I, ■: Location II, ◆: Location III, ✦: Location IV, ●: Spring (SP), ●: Summer (SU), ●: Autumn (A), ●: Winter (W))

The discriminant correspondence analysis (DCA) conducted on the biomass data of the dominant functional groups of phytoplankton reveals that the sorting axis length is below 3 in Lake Chaohu. Consequently, RDA is performed to investigate the relationship between the dominant functional groups of phytoplankton and environmental factors. The results indicate that the eigenvalues of the first two axes of the dominant functional group of phytoplankton are 0.21 and 0.12, respectively. These two axes account for 76.03% of the variance in the relationship between the dominant functional group of phytoplankton and environmental factors (Supplementary Table 3). Seasonal dynamics of dominant functional groups of phytoplankton in Lake Chaohu are obvious (Fig. 7). Functional groups D, N, J, and X3 are dominant in spring, whereas functional groups M and H2 are dominant function groups in summer and autumn. Functional groups J, M, H2, G, and MP are all positively correlated with WT (*P* < 0.05), whereas functional groups X3 and Y are positively correlated with TN, N/P, and SD (*P* < 0.05). In addition, functional group M is positively correlated with DTP, WD, pH, and zooplankton (*P* < 0.05), whereas functional group C is positively correlated with TN and N/P (*P* < 0.01).

Spatially, functional groups M, H2, and G at the biomass level of Location I are significantly higher than those of the other three locations (Fig. 7, *P* < 0.01) and dominate in summer and autumn, showing a positive correlation with water temperature and dissolved total phosphorus. Functional group C dominates in spring and winter. Functional groups C, D, W1, X3, and N dominate significantly in Locations I, II, and III (*P* < 0.001), which are all positively correlated with TN, N/P, and DO. In Location III, J is the dominant functional group in winter and spring. VPA shows that the explanation rate of nutrients to phytoplankton functional groups is 11.5%, whereas environmental factors and zooplankton account for 40.7% and 2.2%, respectively (Fig. 7b). Generally, WT, zooplankton, SD, and TP are the main factors of tempo-spatial variations of dominant functional groups in Lake Chaohu, and their interpretation rate to dominant phytoplankton function groups is 15% (*F* = 8.1, *P* = 0.002), 7.3% (*F* = 4.2, *P* = 0.002), 6.2% (*F* = 3.8, *P* = 0.002), and 4.6% (*F* = 2.9, *P* = 0.016), respectively.

## Discussion

### Historical succession of phytoplankton community structure in Lake Chaohu

In eutrophic lakes, phytoplankton community structures are often mainly composed of Chlorophyta-Cyanophyta or Bacillariophyta-Chlorophyta (Liu et al. 2019; Zhou et al. 2021). The dominant groups of phytoplankton gradually evolved from Chlorophyta to Bacillariophyta in a gap of almost 30 years in Lake Skadar (Rakočević 2012, 2018). A total of 277 species, 85 genera, and 8 phyla are recorded in Lake Chaohu in the early 1980s (Liu and Meng 1988), and the dominant phyla are Chlorophyta, Cyanophyta, and Bacillariophyta, in which this pattern is consistent with subsequent investigations in Lake Chaohu (Deng et al. 2007; Jiang et al. 2014). In this study, the species number of Chlorophyta accounts for 49.47%, followed by Bacillariophyta species (20.53%) and Cyanophyta (13.68%) for the first time. In Lake Chaohu, seasonal fishing is executed before January 2020, whereas comprehensive fishing ban within 10 years have been implemented from January 2020. This change of fishing mode may be an important reason resulting in the change of phytoplankton community structure in Lake Chaohu.

Phytoplankton density and biomass in aquatic ecosystems are affected by nutrient levels (RakočevIć 2018; Haque et al. 2021) and predators (Napiórkowska-Krzebietke 2017). In Lake Chaohu, the changes on the density and biomass of phytoplankton in the past 40 years are shown in Table 3. The annual average density of phytoplankton in 2020–2021 is higher than that in 2011–2012 (Jiang et al. 2014), but lower than that in 2002–2003 (Deng et al. 2007) and 1984 (Liu and Meng 1988). Moreover, the annual average biomass of phytoplankton in 2020–2021 is significantly lower than that in 2002–2003. These results indicate that great variations of phytoplankton density and biomass have occurred in Lake Chaohu since the 1980s, in which it is consistent with changes of fish yield and structure (Wang et al. 2014; Liang et al. 2022). Therefore, it is likely that overfishing of fish increases phytoplankton productivity in 2011–2012 whereas increasing fish yield dropped phytoplankton density and biomass in Lake Chaohu after the fishing ban of 2020. In Lake Donghu, increasing fish production results in the disappearance of cyanobacterial bloom (Liu and Xie 1999). In Lake Warniak, increasing phytophagous fish biomass leads also to the decrease of phytoplankton biomass (Napiórkowska-Krzebietke 2017). However, in Lake Chaohu, the annual average density of phytoplankton has been higher in the western zone of the lake than that in the eastern zone, and *Microcystis* and *Dolichospermum* are dominant species since the 1980s (Liu and Meng 1988; Deng et al. 2007; Jiang et al. 2014; this study). In this study, the phytoplankton density and biomass in Location I are higher than those in the other three locations, in which it locates in the sewage estuary of Paihe River, Shiwuli River, and Nanfei River, and cyanobacterial blooms occur severely (Deng et al. 2007; Jiang et al. 2014; Zhong et al. 2019). Therefore, this evolutionary pattern with unchanged spatial distribution and dominant groups of phytoplankton may be related to long-term eutrophication of Lake Chaohu.

**Table 3:** Historial changes of phytoplankton density (× 10^6^ cells/L) and biomass in Lake Chaohu

Year	Season	Cyano	Bacil	Chlor	Eugle	Crypt	Density	Biomass	Reference
1984	Winter	0.26	0.73	0.06	0.03	0.002	1.08	-	Liu and Meng (1988)
	Spring	0.66	0.012	0.015	0.003	0	0.69	-	
	Summer	48.26	0.001	0.11	0.0002	0.003	48.36	-	
	Autumn	393.79	0.02	0.39	0.01	0.16	394.38	-	
Mean value							394.38		
1987–1988	Winter	0.84	1.01	0.52	0.004	0.67	3.04	-	Tu et al. (1990)
	Spring	3.35	0.10	0.44	0.05	2.25	6.18	-	
	Summer	75.39	0.13	0.20	0.001	0.84	76.56	-	
	Autumn	54.22	0.28	0.36	0	1.38	56.01	-	
Mean value							35.45		
2002–2003	Winter	37.00	7.08	12.71	0.002	1.00	57.94	8.45	Deng et al. (2007)
	Spring	47.81	0.17	1.65	0.002	0.49	50.94	9.79	
	Summer	26.24	1.94	3.49	0.02	0.45	32.15	12.35	
	Autumn	107.70	0.46	1.41	0.008	0.31	109.89	9.84	
Mean value							62.73	10.11	
2011–2012	Winter	-	-	-	-	-	1.94	-	Jiang et al. (2014)
	Spring	-	-	-	-	-	6.37	-	
	Summer	-	-	-	-	-	9.15	-	
	Autumn	-	-	-	-	-	9.12	-	
Mean value							6.65		
2020–2021	Winter	0.88	1.63	2.02	0.02	0.53	5.47	2.24	This study
	Spring	4.27	0.67	2.94	0.02	0	7.92	1.38	
	Summer	178.93	0.99	1.56	0.04	0.77	182.48	10.91	
	Autumn	6.27	1.10	2.05	0.06	0.09	9.75	2.15	
Mean value							51.41	4.80	

Cyano: Cyanophytes; Bacil: Bacillariophytes; Chlor: Chlorophytes; Eugle: Euglenophytes; Crypt: Cryptophytes. "-": No data. Other phytoplankton phylum has not been shown because of very low density

### Influencing factors of temporal-spatial variation of dominant functional groups of phytoplankton

Phytoplankton growth in aquatic ecosystems is limited by various factors (such as water temperature, nutritional level, and light availability) (Varol 2019). Consequently, the dynamics of phytoplankton community structure and functional groups are closely linked to environmental factors (Crossetti et al. 2013; Varol 2019; Kim et al. 2020; Latinopoulos et al. 2020; Ma et al. 2022). In this study, both RDA and VPA show that environmental factors affect temporal and spatial variations of dominant functional groups of phytoplankton in Lake Chaohu. The representative species of functional group J are *Pediastrum* sp. and *Desmodesmus quadricauda* in spring, whose habitat type is turbid and shallow eutrophic water and resistant to low light and low temperature (Reynolds et al. 2002, 2006; Padisák et al. 2006, 2009). The dominant functional group D only appears in Location II in spring, which is consistent with the low temperature, low light, and mesotrophic environment in Lake Chaohu, and it also occurs in other waterbodies (Reynolds et al. 2002, 2006; Padisák et al. 2006; Silva et al. 2018). High temperature, high phosphorus, and light are conducive to the growth of dominant functional group M, resulting in the outbreak of cyanobacterial blooms in summer and autumn in Lake Chaohu, in which this phenomenon appears in other eutrophic waterbodies (Yoshinaga et al. 2006; Cao et al. 2018). Moreover, in this study, the relative biomass of dominant function group M in Location I is higher than that in the other three locations (Locations II, III, and IV), in which it relates to a large number of nutrients and exogenous organic substances from Shiwuli River, Nanfei River, and Paihe River (Jiang et al. 2014; Zhong et al. 2019). In some researches, functional group H2 grows well under low nitrogen, low light, and high phosphorus, and its biomass decreases with rising water levels (Padisák et al. 2006; Yang et al. 2016; Nan et al. 2020). In this study, the relative biomass of dominant functional group H2 rises in autumn and occupies a higher proportion in Location IV. Furthermore, function group MP is mainly composed of Bacillariophyta species in spring but Cyanophyta species in autumn with increasing water temperature and nutrient concentrations; similarly, research findings have been reported in other aquatic ecosystems such as Lake Luoma, Lake East Taihu, and Daning River (Zhu et al. 2013; Tian et al. 2018; Nan et al. 2020). In this study, the dominant functional groups M, H2, MP, and J are all positively correlated with water temperature (WT), and RDA also shows that WT (15%) has the highest interpretation rate of phytoplankton functional groups, indicating that WT is a crucial limiting factor for the dynamics of phytoplankton functional groups in Lake Chaohu. Similar observations have been reported in Lake Santa Lucia, Lake East Taihu, and Lake Okeechobee (Silva et al. 2018; Nan et al. 2020; Ma et al. 2022). In Lake Chaohu, function groups Y and N are dominant in winter, and they are related to low temperature and high turbidity, consistent with the findings of other studies (Xu et al. 2011; Nan et al. 2020). Therefore, environmental heterogeneity is identified as one of the key factors influencing the temporal variations and spatial distributions of dominant functional groups of phytoplankton in Lake Chaohu.

Fish predation can have a significant impact on the structure and dynamics of phytoplankton communities, both directly and indirectly through top-down effects (Attayde and Hansson 1999). On the one hand, grazing pressure by *H. molitrix* and *A. nobilis* may directly affect phytoplankton abundance and biomass (Vörös et al. 1997; Roozen et al. 2007). On the other hand, it may also indirectly affect phytoplankton structure and biomass by suppressing zooplankton biomass (Jeppesen et al. 2003; Shen et al. 2021). The density and species of fish in Lake Chaohu show a decreasing trend, and the dominant species gradually turned into small-sized species over the past 50 years (Deng et al. 2007; Liang et al. 2022). However, with the implementation of the 10-year fishing ban in Lake Chaohu on January 2020, fish predation pressure on both zooplankton and phytoplankton is expected to greatly enhance fish predation pressure on both zooplankton and phytoplankton, because some small zooplankton-feeding fishes, such as *C. ectenes* and *Neosalanx tangkahkeii*, primarily feed on zooplankton, while omnivorous fishes such as *H. molitrix* and *A. nobilis* feed on zooplankton and phytoplankton (Mao et al. 2011; Liang et al. 2022). In this study, the dominant functional group J, which includes *Pediastrum biradiatum*, *Scenedesmus* sp., and *Desmodesmus quadricauda* in spring, is found to be related to lower zooplankton densities. This suggests that fish predation pressure indirectly contributes to the community structure and temporal-spatial dynamics of phytoplankton.

## Conclusion

In this study, the cell density, biomass, and dominant functional groups of phytoplankton exhibited significant temporal-spatial variations in Lake Chaohu. Temporally, the lowest biomass is observed in spring, with dominant functional groups D, N, J, M, and X3. In contrast, both cell density and biomass of phytoplankton are highest in summer, with M and H2 being the main functional groups. The density and biomass of phytoplankton decrease in autumn, in which dominant functional groups C, G, and MP co-exist with H2 and M. In winter, the dominant functional groups of phytoplankton in the lake converted to C, N, Y, and T. Spatially, phytoplankton density and biomass in Location I are higher than those in the other three locations. The maximum relative biomass dominant functional groups M, H2, and C appear in Location I, Location III, and Location IV, respectively. All thirteen dominant functional groups occur in Location II. RDA and VPA reveal that WT, zooplankton, SD, and TP are main factors affecting the temporal-spatial variations of dominant functional groups in Lake Chaohu. Our results suggest that spatial distributions of dominant functional groups of phytoplankton are affected by environmental heterogeneity in Lake Chaohu.

## Supplementary Information

Below is the link to the electronic supplementary material.

Supplementary file1 (DOCX 39 KB)

## Data Availability

The environmental factor dataset and phytoplankton dataset are available from the corresponding author (dengdg@chnu.edu.cn) upon reasonable request.
