# Supplementary material for: Environmental heterogeneity affecting spatial distribution of phytoplankton community structure and functional groups in a large eutrophic lake, Lake Chaohu, China
Source: Environ Sci Pollut Res Int. 2023 Jun 6;30(32):79001–14. doi: 10.1007/s11356-023-28043-5 (PMC10313546; doi:10.1007/s11356-023-28043-5)
Supplement: Supplementary file 1 — Supplementary file1 (DOCX 39 KB) [file 11356_2023_28043_MOESM1_ESM.docx]

Supplementary Table 1 Two-way ANOVA on physo-chemical parameters and phytoplankton density and biomass based on temporal and spatial variations in Lake Chaohu

| Parameters | *df* | *F* | *P* |
| --- | --- | --- | --- |
| Water temperature (℃) |  |  |  |
| Season | 3 | 203.64 | <0.001 |
| Location | 3 | 0.096 | >0.05 |
| Season×Location | 9 | 0.089 | >0.05 |
| Dissolved oxygen (mg/L) |  |  |  |
| Season | 3 | 49.792 | <0.001 |
| Location | 3 | 3.436 | <0.05 |
| Season×Location | 9 | 1.844 | >0.05 |
| Total nitrogen (mg/L) |  |  |  |
| Season | 3 | 30.94 | <0.001 |
| Location | 3 | 32.394 | <0.001 |
| Season×Location | 9 | 0.245 | >0.05 |
| Total phosphorus (mg/L) |  |  |  |
| Season | 3 | 13.762 | <0.001 |
| Location | 3 | 5.719 | 0.001 |
| Season×Location | 9 | 2.37 | 0.014 |
| Dissolved total phosphorus (mg/L) |  |  |  |
| Season | 3 | 13.228 | <0.001 |
| Location | 3 | 2.483 | >0.05 |
| Season×Location | 9 | 0.66 | >0.05 |
| N/P |  |  |  |
| Season | 3 | 2.483 | >0.05 |
| Location | 3 | 8.021 | <0.001 |
| Season×Location | 9 | 1.167 | >0.05 |
| Water depth (m) |  |  |  |
| Season | 3 | 33.152 | <0.001 |
| Location | 3 | 33.175 | <0.001 |
| Season×Location | 9 | 0.921 | 0.921 |
| Transparency (m) |  |  |  |
| Season | 3 | 8.683 | <0.001 |
| Location | 3 | 1.157 | >0.05 |
| Season×Location | 9 | 2.096 | 0.031 |
| pH |  |  |  |
| Season | 3 | 11.204 | <0.001 |
| Location | 3 | 6.522 | <0.001 |
| Season×Location | 9 | 1.688 | >0.05 |
| Phytoplankton density (×10^7^cells/L) |  |  |  |
| Season | 3 | 65.009 | <0.001 |
| Location | 3 | 6.833 | <0.001 |
| Season×Location | 9 | 1.923 | 0.05 |
| Phytoplankton biomass (mg/L) |  |  |  |
| Season | 3 | 44.716 | <0.001 |
| Location | 3 | 11.927 | <0.001 |
| Season×Location | 9 | 2.265 | 0.019 |

Supplementary Table 2 Phytoplankton species and seasonal distribution in Lake Chaohu during 2020-2021

| Species | Spring | Summer | Autumn | Winter |
| --- | --- | --- | --- | --- |
| **Chlorophyta** |  |  |  |  |
| *Chlamydomonas* sp. | + | - | + | + |
| *C. globosa* Snow | + | - | + | + |
| *C. stellata* Dill | + | - | + | + |
| *Pteromonas* sp. | + | + | + | + |
| *Micractinium pusillum* Frasenius | + | + | + | + |
| *Golenkinia radiata* Chodat | + | + | + | + |
| *Errerella bornhemiense* Conrad. | - | - | + | - |
| *Trochisia reticularis* (Reinsch) Hansgirg | - | + | + | + |
| *Eudorina elegans* Ehr.^abc^ | + | + | + | + |
| *Pandorina morum* (Müll) Bory | + | + | + | + |
| *Sphaerocystis schroeteri* Chodat | - | - | + | - |
| *Gloeoactinium limneticum* G. M. Smith | - | - | + | + |
| *Schroederia robusta* Korschikoff | + | + | + | + |
| *S. nitzschioides* (G. S. West) Korschikoff | + | + | + | + |
| *S. spiralis* (Printz) Korschikoff | + | + | + | + |
| *Schroederia* sp. | + | + | + | + |
| *Chlorella vulgaris* Beijerinck | + | + | + | + |
| *Selenastrum* sp. | + | + | + | + |
| *S. minutum* (Näg.) Collinus | + | + | + | + |
| *S. westii* G. M. Smith | + | + | + | + |
| *Kirchneriella* sp. | + | + | + | + |
| *K. obesa* (W. West) Schmidle | + | + | + | + |
| *Ankistrodesmus falcatus* (Corda) Ralfs | + | + | + | + |
| *A. angustus* Bernard | + | + | + | + |
| *A. acicularis* (A. Braun) Korschikoff | + | + | + | + |
| *A. convolutus* Corda | + | + | + | + |
| *A. falcatus* var. *mirabilis* (West & West) G. S. West | + | + | + | + |
| *Closteriopsis longissima* (Lemmermann) Lemmermann | + | + | + | + |
| *Oocystis* sp. | + | + | + | + |
| *O. lacustris* Chodat | + | + | + | + |
| *O. borger* Snow^a^ | + | + | + | + |
| *Nephrocytium* sp. | + | + | + | + |
| *Quadrigula chodatii* (Tann.-Fuiim.) G. M. Smith | + | + | + | + |
| *Actinastrum fluviatile* (Schroed) Fott, Preslia | + | + | + | + |
| *Dictyosphaerium pulchellum* Wood | + | + | + | + |
| *Dictyosphaerium* sp. | + | + | - | - |
| *Tetraedron* sp. | + | + | + | + |
| *T. bi furcatum* (Wille) Lagerheim | + | + | + | - |
| *T. trigonum* (Näg.) Hansgirg | + | + | + | + |
| *T. trilobulatum* (Reinsch) Hansgirg | + | + | + | + |
| *T. caudatum* (Corda) Hansgirg | + | + | + | + |
| *T. minimum* (A. Braun) Hansgirg | + | + | + | + |
| *Pediastrum duplex* Meyen | + | + | + | + |
| *P. simples* Meyen | + | + | + | + |
| *P. simplex* var. *duodenarium* (Bail.) Rabenhorst | + | + | + | - |
| *P. boryannum* (Turp.)Meneghini | + | + | + | + |
| *P. tetras* (Ehr.) Ralfs | + | + | + | + |
| *P. duplex* var. *gracillimum* West & West | + | + | + | - |
| *P. biradiatum* Meyen | + | + | + | + |
| *P. tetras* var. *tetraodon* (Corda) Rabenhorst | + | + | + | + |
| *Scenedesmus* sp. | + | + | + | + |
| *S. javaensis* Chodat | + | + | + | + |
| *S. obliquus* (Turp.) Kützing | + | + | + | + |
| *S. arcuatus* Lemmermann | + | + | + | + |
| *S. bijuga* (Turp.) Kützing | + | + | + | + |
| *S. arcuatus* var. *platydiscus* (G. M. Smith) Chodat | - | + | - | - |
| *S. acuminatus* (Lag.) Chodat | + | + | + | + |
| *Acutodesmus. dimorphus* (Turpin) Tsarenko | + | + | + | + |
| *Desmodesmus quadricauda* (Turpin) Hegewald^a^ | + | + | + | + |
| *D. denticulatus s* (Lagerheim) An, Friedl & Hegewald | + | + | + | + |
| *D. perforatus* (Lemmermann) Hegewald | - | + | + | - |
| *Ulothrix* sp. | + | - | + | + |
| *Crucigenia* sp. | + | + | + | + |
| *C. quadrata* Morren | + | + | + | + |
| *C. tetrapedia* (Kirchn.) West & West | + | + | + | + |
| *C. lauterbornii* Schmidle | + | + | + | - |
| *C. apiculata* (Lemm.) Schmidle | + | + | + | + |
| *Chodatella* sp. | - | + | + | - |
| *C. quadriseta* Lemmermann | + | + | + | + |
| *C. wratislaviensis* (Schroed.) Ley | + | - | + | + |
| *Coelastrum microporum* Nägeli^a^ | + | + | + | + |
| *C. sphaericum Nägeli* | + | + | + | + |
| *C. proboscideum* Bohlin | + | + | + | - |
| *Closterium* sp. | + | + | + | + |
| *C. venus* Kützing | + | + | + | + |
| *Staurastrum* sp. | + | + | + | + |
| *Cosmarium* sp.^ad^ | + | + | + | + |
| *C. circulare* Reinsch | + | + | + | + |
| *C. depressum* (Näg.) Lundell | + | - | + | + |
| *Staurodesmus* sp. | + | + | + | + |
| *Gonatozygon* sp. | - | + | + | + |
| *Tetrastrum* sp. | + | + | + | + |
| *T. heterocanthum* (Nordst.) Chodat | + | + | + | + |
| *T. staurogeniae forme* (Schroed.) Lemmermann | + | + | + | + |
| *T. elegans* Playfair | + | + | + | + |
| *Treubaria. crassispina* G. M. Smith | + | + | + | + |
| *Carteria globulosa* Pascher | + | - | + | + |
| *C. klebsii* (Dang) France em. Troitzk | + | - | + | + |
| *Wislouchiella* sp. | + | + | + | + |
| *Franceia ovalis* (France) Lemmermann | + | + | + | + |
| *Tetrallantos* sp. | - | + | + | + |
| *Echinosphaerella* sp. | + | + | + | - |
| *Tetradesmus wisconsinense* G. M. Smith | + | + | + | + |
| *Cladophora* sp. | - | - | - | + |
| **Cryptophyta** |  |  |  |  |
| *Chroomonas acuta* Uterm | + | + | + | + |
| *C. caudata* Geitler | + | + | + | + |
| *Cryptomonas ovata* Ehr.^d^ | + | + | + | + |
| *C. erosa* Ehr.^d^ | + | + | + | + |
| **Dinophyta** |  |  |  |  |
| *Ceratium hirundinella* (O.F. Müller) Dujardin | + | + | + | + |
| *Gymnodinium aeruginosum* Stein | + | + | + | + |
| *Peridinium* sp. | + | + | + | + |
| *P. umbonatum* Stein | - | + | + | - |
| **Chrysophta** |  |  |  |  |
| *Dinobryon cylindricum* Imhof, ex Ahlstrom | + | - | - | + |
| *D. bavaricum* Imhof | + | - | - | + |
| *Ochromonas* sp. | + | + | + | - |
| *Chromulina* sp. | + | + | + | + |
| *C. elegans* Doflein | + | + | + | + |
| *Synura nvella* Ehrenberg, em. Korshikov | + | - | - | + |
| **Bacillaripohyta** |  |  |  |  |
| *Melosira granulata* (Ehr.) Ralfs^abd^ | + | + | + | + |
| *M. varians* Agardh | + | + | + | - |
| *M. islandica* O. Müller | + | + | + | + |
| *M. italica* (Ehr.) Kützing | + | + | + | + |
| *M. granulata* var. *angustissima f. spiralis* Hustedt | + | + | + | - |
| *M. granulata* var. *angustissima* O. Müller^d^ | + | + | + | + |
| *Cyclotella* sp.^ad^ | + | + | + | + |
| *C. meneghiniana* Kützing^abcd^ | + | + | + | + |
| *C. stelligera* (Cleve & Grunow) | + | + | + | + |
| *C. hubeiana* Chen & Zhu | + | + | + | + |
| *C. kuetzingiana* Thwaites | + | + | + | + |
| *Tabellaria fenestrata* (Lyngb.) Kützing | + | - | + | - |
| *Asterionella formosa* Hassall | + | - | - | + |
| *Fragilaria* sp. | + | + | + | + |
| *F. capucina* Desmaziéres | + | + | + | + |
| *Cymbella* sp. | + | + | + | + |
| *C. tumida* (Bréb. ex Kütz.) Van Heurck | - | - | + | + |
| *C. perpusilla* Cleve | + | - | - | + |
| *Synedra* sp. | + | + | + | + |
| *S. acus* Kützing | + | + | + | + |
| *S. affinis* Kütz | + | + | + | + |
| *S. ulna* (Nitzsch.) Ehrenberg | + | + | + | - |
| *Achnanthes* sp. | + | + | + | + |
| *Navicula* sp. | + | + | + | + |
| *Frustulia* sp. | - | - | + | + |
| *Gomphonema* sp. | + | + | - | + |
| *G. olivaceum* (Lyngbye) Kützing | + | - | - | + |
| *Pinnularia* sp. | + | + | + | + |
| *Stauroneis anceps* Ehrenberg | + | + | + | + |
| *Gyrosigma acuminatum* (Kütz.) Rabenhorst | + | - | + | + |
| *Cocconeis placentula* Ehrenberg | + | - | + | + |
| *Surirella* sp. | - | + | + | + |
| *Nitzschia* sp. | - | - | - | + |
| *N. linearis* W. Smith | + | + | - | + |
| *Amphora Ehrenberg ovalis* (Kütz.) Kützing | + | - | + | + |
| *Diatoma vulgare* Borger | + | + | - | + |
| *Coscinodiscus excentricus* Ehrenberg^ad^ | + | + | + | + |
| *Stephanodiscus* sp. | - | + | + | + |
| *Urosolenia* sp. | + | + | + | + |
| **Euglenophyta** |  |  |  |  |
| *Euglena* sp. | + | + | + | + |
| *E. pisciformis* Kleds | + | + | + | + |
| *E. acus* Ehrenberg | + | + | + | - |
| *E. mutabilis* Schmitz | + | - | + | - |
| *Phacus* sp. | + | + | + | + |
| *P. tortus* (Lemm.) Skv. | + | + | + | + |
| *P. longicauda* (Ehr.) Duj. | + | + | + | + |
| *P. cylindrus* Pochm. | + | + | + | + |
| *P. pyrum* (Ehr.) Stein | + | + | - | + |
| *Trachelomonnas* sp. | + | + | + | + |
| *T. oblonga* Lemm | + | - | + | + |
| *Lepocinclis* sp. | + | + | + | + |
| *Strombomonas* sp. | + | + | + | + |
| *S. ensifera* (Dad.) Defl. | + | + | + | + |
| **Xanthophyta** |  |  |  |  |
| *Tribonema* sp.^d^ | + | + | + | + |
| *T. minus* (Will.) Haz.^d^ | + | + | + | + |
| *Goniochloris* sp. | - | - | - | + |
| **Cyanophyta** |  |  |  |  |
| *Dactyloccocopsis rhaphidioides* Lemm. | + | + | + | + |
| *Merismopedia minima* G. Beck | + | + | + | - |
| *M. tenuissima* Lemm. | + | + | + | + |
| *Chroococcus* sp. | + | + | + | + |
| *Aphanocapsa elachista* W. et. G. S. West | + | + | + | + |
| *Spiralina* sp. | + | + | + | - |
| *Phormidium* sp. | - | - | + | - |
| *P. tenue* (Menegh.) Gom. | + | + | + | + |
| *Oscillatoria* sp.^acd^ | + | + | + | + |
| *O. subbrevis* Schm. | - | + | + | - |
| *O. acuminata* Gom. | + | + | + | - |
| *Aphanizomenon flos-aquae* (L.) Ralfs.^d^ | + | + | + | + |
| *Raphidiopsis sinensia* Jao | + | + | + | + |
| *Pleurocapsa fuliginosa* Hauck. | + | + | + | + |
| *Lyngbya* sp. | + | + | + | + |
| *Synechocystis aquetilis* Sauvageau | + | + | - | + |
| *Aphanothece* sp. | - | - | + | + |
| *Nostoc* sp. | - | + | - | + |
| *Pseudanabaena mucicola* (Naumann & Huber-Pestalozzi) Schwabe | + | + | + | + |
| *Dolichospermum* sp.^bc^ | + | + | + | + |
| *D. flos-aquae (*Brébisson ex Bornet & Flauhault) Wacklin, Hoffmann & Komárek^bc^ | + | + | + | + |
| *Microcystis* sp.^bc^ | + | + | + | + |
| *M. wesenbergii* (Komárek) Komárek | - | - | + | - |
| *M. aeruginosa Kützing*^bc^ | + | + | + | - |
| *M. flos-aquae* (Wittrock) Kirchner^bc^ | + | + | + | - |
| *M. ichthyoblabe* Kütz. | - | + | + | + |

“**+**”: present; “-”: not present. a b c and d: represent the dominant species (*Y*>0.02) in spring, summer, autumn and winter, repspectively.

Supplementary Table 3 Redundancy analysis between dominant functional groups of phytoplankton and environmental factors in Lake Chaohu

| Axes | 1 | 2 | 3 | 4 | Total inertia |
| --- | --- | --- | --- | --- | --- |
| Eigenvalues | 0.214 | 0.1236 | 0.050 | 0.026 | 1.000 |
| Species-environment correlations | 0.754 | 0.716 | 0.747 | 0.641 |  |
| Cumulative percentage variance of species data | 27.06 | 43.86 | 53.72 | 62.44 |  |
| Species-environment relation | 48.19 | 76.03 | 87.37 | 93.26 |  |
| Test of Monte Carlo permutation Variable: | *F*=3.000, *P*=0.002 | | | | |
